# Sulfuric Acid Immobilized on Activated Carbon Aminated with Ethylenediamine: An Efficient Reusable Catalyst for the Synthesis of Acetals (Ketals)

**DOI:** 10.3390/nano12091462

**Published:** 2022-04-25

**Authors:** Wenzhu Liu, Ruike Guo, Guanmin Peng, Dulin Yin

**Affiliations:** 1 College of Chemistry and Materials Engineering, Huaihua University, Huaihua 418000, China; hebeiliuwenzhu@126.com (W.L.); guanminpengh@126.com (G.P.); 2 National & Local Joint Engineering Laboratory for New Petro-Chemical Materials and Fine Utilization of Resources, Hunan Normal University, Changsha 410081, China

**Keywords:** activated carbon, solid acid catalyst, benzaldehyde, acetals (ketals)

## Abstract

Through the amination of oxidized activated carbon with ethylenediamine and then the adsorption of sulfuric acid, a strong carbon-based solid acid catalyst with hydrogen sulfate (denoted as AC-N-SO_4_H) was prepared, of which the surface acid density was 0.85 mmol/g. The acetalization of benzaldehyde with ethylene glycol catalyzed by AC-N-SO_4_H was investigated. The optimized catalyst dosage accounted for 5 wt.% of the benzaldehyde mass, and the molar ratio of glycol to benzaldehyde was 1.75. After reacting such mixture at 80 °C for 5 h, the benzaldehyde was almost quantitatively converted into acetal; the conversion yield was up to 99.4%, and no byproduct was detected. It is surprising that the catalyst could be easily recovered and reused ten times without significant deactivation, with the conversion yield remaining above 99%. The catalyst also exhibited good substrate suitability for the acetalization of aliphatic aldehydes and the ketalization of ketones with different 1,2-diols.

## 1. Introduction

Synthesis of acetals (ketals) are a class of reactions that are widely used in various fields, such as organic synthesis [1,2], medical materials [3], carbohydrate chemistry [4], and bio-based solvents [5]. As a typical representative, benzaldehyde condensed with ethylene glycol has attracted considerable interest in a number of studies and is widely used as flavors due to its properties of a fruity aroma with an apple flavor, long-lasting fragrance, and good chemical stability [6,7]. In traditional catalytic syntheses, sulfuric acid, hydrochloric acid, p-toluenesulfonic acid, and other inorganic acids can be used as catalysts in synthesizing acetals (ketals) reactions [8]. These catalytic processes have advantages of simplicity and high conversion efficiency but are also accompanied by the disadvantages of side reactions, difficulties in products separation, and the corrosion of equipment. Therefore, it is imperative to find an appropriate solid acid alternative to traditional liquid acid catalysts. Currently, molecular sieves [9], solid superacid [10], heteropoly acid (heteropoly-acid-based ionic liquids [11], TiO_2_ nanoparticle-exfoliated montmorillonite [12], 8-hydroxy-2-methylquinoline-modified with H_4_SiW_12_O_40_ [13], Ta/W mixed addenda heteropolyacid [14], solid oxide acid [7], and carbon-based solid acid [15] have been used to catalyze the synthesis of acetals (ketals), achieving good catalytic effects but also accompanied by low yield, poor selectivity, difficult recovery, poor solvent applicability, and the loss of active components [15,16,17]. Thus, novel economical, efficient, and reusable solid acid catalysts used in the synthesis of acetals still need to be developed. For the past few years, solid carbon-based catalysts have attracted the attention of researchers due to the advantages of abundant resources, large specific surface areas, easy-to-control pore structures, and abundant controllable aromatic rings and oxygen-containing functional groups on the surface [18,19]. The catalytic performances of carbon materials are inseparably related to the type of surface acidic or basic functional groups. Acidic groups obtained by the simple oxidation of carbon with oxidants, such as HNO_3_ [20,21], H_2_SO_4_ [22], H_2_O_2_ [23], and other oxidants [24], are usually weak acidic groups, such as hydroxyl, carboxyl, etc., which stimulate the use in absorption and desorption fields [25,26] but cannot be applied satisfactorily in the field of catalysis due to the strong acidic requirements of catalytic reactions. However, the abundant oxygen-containing functional groups on the surface of carbon provided many possibilities for designing surface functional groups and use in different catalytic systems. Graphene, mesoporous carbons, and activated carbon are carbon materials that can be equipped with strong acidic catalytic groups on their surface and as such have been synthesized with different pretreatment methods and applied in the synthesis of acetals (ketals) [27,28,29,30,31]. Hosseini M.S. [27] prepared a SulAmp-AC catalyst with the chemically attached sulfonic acid groups after surface modification with a suitable nitrogen-containing spacer group on AC; the conversion rate for benzaldehyde was 98% when the prepared SulAmp-AC used as catalyst. When propyl-SO_3_H functionalized graphene oxide (GO-PrSO_3_H) modified with (3-mercaptopropyl) trimethoxysilane and the thiol groups oxidized to surface -SO_3_H residues was used as catalyst, the conversion of benzaldehyde was 92% [28]. Yuan C. [29] synthesized sulfonic-acid-functionalized core-shell Fe_3_O_4_@carbon microspheres (Fe_3_O_4_@C-SO_3_H), and the conversion of benzaldehyde was 69% when it was used as a catalyst. Although the different carbon-based catalysts that have been studied exhibit good catalytic effects on synthesizing acetals (ketals), its poor reusability due to the leaching of surface-active functional groups [29,31] is still the main disadvantage of strong carbon-based solid catalysts. The exploration of methods for preparing stable carbon-based acid catalysts with excellent catalytic performance and stable functional groups was the focus of related research. 

In the preliminary work, our group successfully attached stable aminated groups on activated carbon with ethylenediamine [32]. Then, to explore carbon materials with strong and stable acidic functional groups, aminated activated carbon was treated through impregnation in aqueous sulfuric acid. The effects of preparation conditions on acidic functional group content on activated carbon surface were investigated. To study catalytic performance and the stability of acidic groups on the activated carbon surface, condensation of benzaldehyde with ethylene glycol was used as a probe reaction. The catalyst reusability and substrate suitability in synthesizing acetals (ketals) reactions were also studied.

## 2. Materials and Methods

### 2.1. AC-N-SO_4_H Preparation

**Figure 1:**
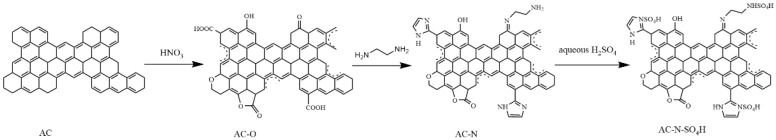
Scheme of preparing AC-N-SO_4_H.

Activated carbon (AC) as a raw material was oxidized with HNO_3_ and aminated with ethanediamine. Details of the amination of AC leading to aminated activated carbon (AC-N) was previously reported [32]. The intermediate was treated with aqueous sulfuric acid to produce the catalyst denoted as AC-N-SO_4_H. The product was isolated by filtration and dried at 105 °C for 24 h. The scheme of AC-N-SO_4_H preparation is shown in Figure 1.

### 2.2. Sample Characterization

The density of acid was measured with back titration method: 50 mg sample was added to 20 mL 0.01 mol/L NaOH and then sonicated for 30 min. After filtration and being washed with distilled water, groups on AC-N-SO_4_H were determined with 0.01 mol/L HCl using mixed bromocresol and green-methyl red as an indicator. FT-IR spectroscopy analysis was performed using Perkin Elmer 283 spectrometer (Perkin Elmer Instruments Co., Ltd., Waltham, MA, USA). The solid was mixed with KBr powder, and the mixture was pressed into pellets to conduct FT-IR analyses. The FT-IR spectra were recorded between 4000 and 400 cm^−1^ with a resolution of 4 cm^−1^ and acquisition rate of 20 scan·min^−1^. In order to analyze the thermal stability of the sample, NETZSCH STA 409 PC/PG (NETZSCH-Gerätebau GmbH, Selb, Germany) thermal gravimetric analyzer was used. The conditions were as follows: Under 10 °C/min heating rate, 20 mg sample was heated from room temperature to 800 °C under N_2_. Using TriStar 3000 surface area analyzer (Micromeritics Instrument Ltd., Atlanta, GA, USA), samples surface properties and surface area were characterized with N_2_ adsorption measurements at 77 K. The surface area (S_BET_) was calculated from isotherms using the Brunauer–Emmett–Teller (BET) equation. The volume of liquid nitrogen corresponding to the amount adsorbed at a relative pressure of P/P_0_ = 0.99 was defined as the total pore volume.

### 2.3. AC-N-SO_4_H Catalytic Properties on Synthesis of Acetals (Ketals)

AC-N-SO_4_H catalytic properties on synthesis of acetal (ketal) reaction were tested. Generally, substrates with certain amounts of AC-N-SO_4_H were added to a three-necked flask, which was equipped with thermometer and condenser. The effects of reaction temperature, reaction time, catalyst dosage, and molar ratio of alcohol/aldehyde on conversion were investigated. The recycling performance and substrate suitability of AC-N-SO_4_H were also studied. Agilent 6890N gas chromatograph (Agilent Technologies Inc., Santa Clara, CA, USA) was used to quantitatively analyze the conversion of benzaldehyde and product selectivity. The analytical conditions were: toluene as the internal standard, SE-30 capillary column (Beijing Huarui Boyuan S&T development Co., Ltd., Beijing, China) (30 m × 0.25 mm × 0.25 μm), high-purity nitrogen as carrier gas with 1.0 mL/min flow rate, FID detector temperature 250 °C, injector temperature 250 °C, column pressure 0.6 MPa, injection volume 0.2 μL. The column temperature was temperature-programmed as: held for 3 min at 100 °C, then increased to 200 °C at a rate of 20 °C/min, and held for 1 min.

## 3. Results and Discussion

### 3.1. AC-N-SO_4_H Preparation

**Figure 2:**
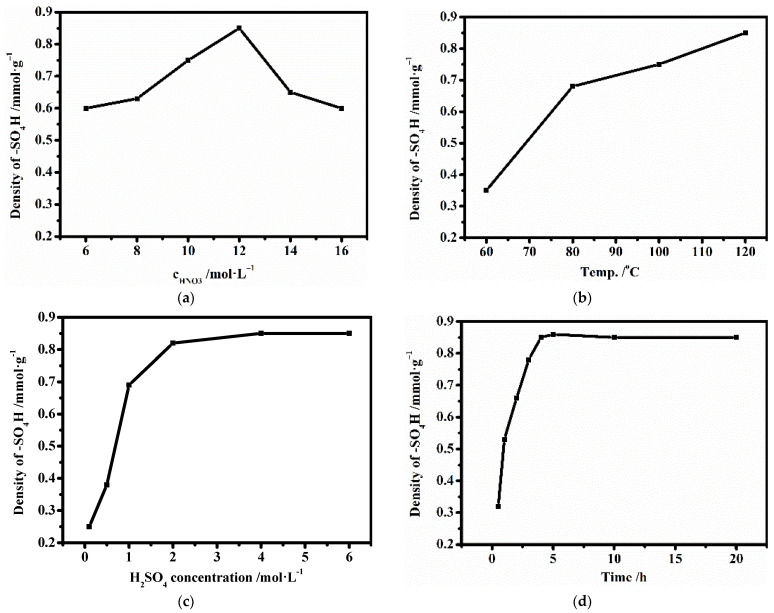
Effect of the preparation of AC-N-SO_4_H on the density of -SO_4_H. (**a**) HNO_3_ concentration; (**b**) amination temperature; (**c**) H_2_SO_4_ concentration; (**d**) impregnation time.

The effects of HNO_3_ concentration in the oxidation process, reaction temperature in the amination process, and dilute aqueous sulfuric acid concentration in the acidification process on the amount of acid on the AC-N-SO_4_H surface were investigated. The typical impregnation procedure was as follows: 1 g of AC-N and 20 mL of 4 mol/L aqueous sulfuric acid were mixed in a beaker and stirred at room temperature for 4 h. After completion, the prepared solid was filtered and washed, then dried at 105 °C for 24 h to prepare AC-N-SO_4_H. The effects of preparation conditions are shown in Figure 2a–d.

As shown, the density of -SO_4_H increased gradually with the initial increased concentration of nitric acid, but when the HNO_3_ concentration exceeded 12 mol/L, the density of -SO_4_H decreased rapidly to 0.5 mmol/L, which was attributed to the reduction of AC surface functionalizable structures due to strong oxidation process. With the increased temperature in amination process, the density of -SO_4_H gradually increased, which indicated that the increase of amination temperature had no destructive effect on AC surface structure as opposed to that of the HNO_3_ concentration. With the increased concentration of aqueous sulfuric acid and longer impregnation time, the density of -SO_4_H gradually increased. At a concentration of 4 mol/L and impregnation time of over 4 h, the density of -SO_4_H did not increase further. In summary, when the HNO_3_ concentration was 12 mol/L, the amination temperature was 120 °C, the dilute aqueous sulfuric acid concentration was 4 mol/L, and impregnation time was 4 h, the maximum density of -SO_4_H was 0.85 mmol/g.

### 3.2. AC-N-SO_4_H Structural Analysis

#### 3.2.1. Specific Surface Area

**Figure 3:**
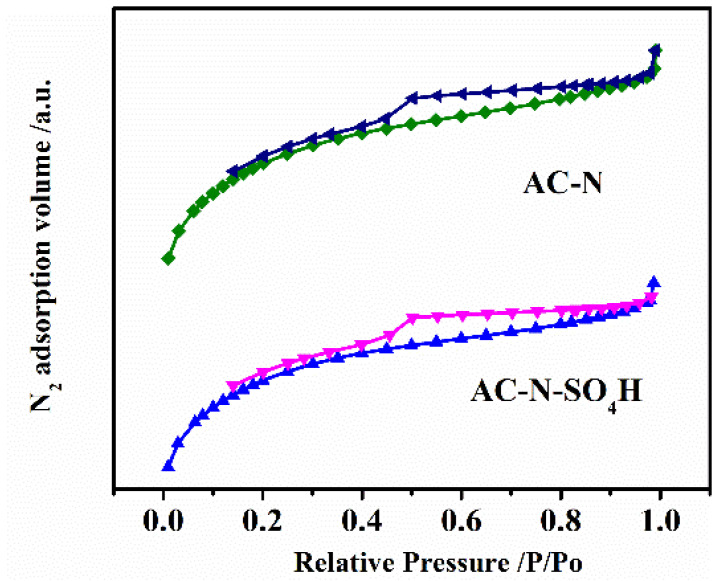
N_2_ adsorption–desorption isotherms for AC-N and AC-N-SO_4_H.

**Figure 4:**
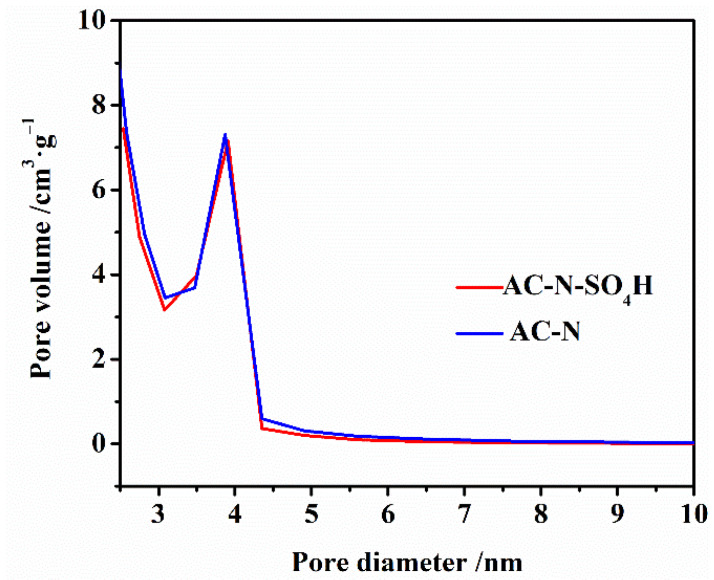
Pore size distribution for AC-N and AC-N-SO_4_H.

The standard BET equation was used to calculate the surface area of AC-N-SO_4_H and its precursor, AC-N. The nitrogen adsorption-desorption curves are shown in Figure 3. The N_2_ adsorption isotherms of the samples were belonged to the type IV class, which indicated the presence of a uniform mesoporous structure [32,33]. An upturned “tail” and obvious hysteresis loop are shown in both adsorption isotherms, indicating that AC-N and AC-N-SO_4_H had a mesoporous structure. Figure 4 shows the results of the pore size distribution measurements for the samples, which indicated that the impregnation process with aqueous H_2_SO_4_ did not destroy the mesoporous structure in AC-N. The porous structures of samples are shown in Table 1. According to Table 1, the BET surface area of AC-N-SO_4_H was 384 m^2^/g, only slightly lower than that of AC-N’s 418 m^2^/g, which illustrated that there were absolutely no detriments to the pore volume and pore size in the acidification process.

#### 3.2.2. FT-IR

**Figure 5:**
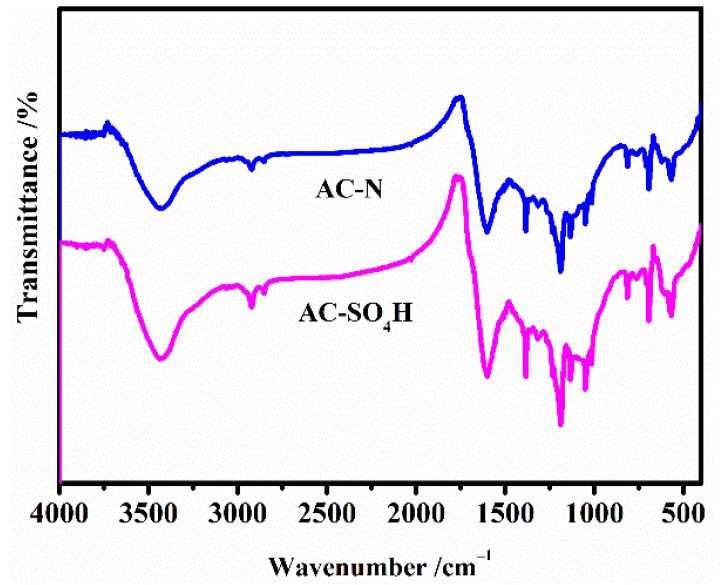
FT-IR spectrum of AC-N-SO_4_H and AC-N.

FT-IR spectra of AC-N-SO_4_H and its precursor AC-N are shown in Figure 5. According to Figure 5, the strong absorption band around 3400 cm^−1^ corresponds to stretching of carboxylic O-H group. The broad absorption peak near 1200 cm^−1^ was the stretching vibration of groups containing single-bonded oxygen atoms or single-bonded nitrogen atoms, including phenolic hydroxyl groups, ether bonds, lactones, CN, -NH, -NH_2_, etc. The absorption peak that appeared at 1604 cm^−1^ was due to CN stretching vibration, and NH stretching vibration, which supposedly appeared at 3400 cm^−1^, almost overlapped with -OH stretching vibration. The FT-IR spectrum of AC-N-SO_4_H almost overlapped with that of AC-N, indicating that the treatment of aminated activated carbon impregnated with sulfuric acid did not destroy the N-containing structure on the AC-N surface. Combined with the analysis of the types and contents of functional groups on the surface of activated carbon, the hydrogen sulfate was successfully grafted on the aminated structure.

#### 3.2.3. TG-DTG

**Figure 6:**
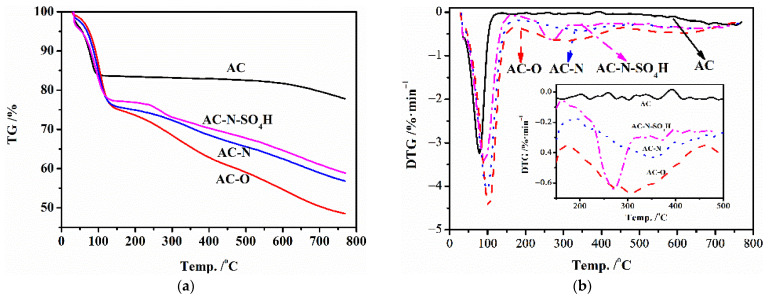
TG and DTG performance of AC-N-SO_4_H and its precursors. (**a**) TG; (**b**) DTG.

Thermogravimetric analysis was used to analyze the thermostability of AC-N-SO_4_H and its precursors; the results are displayed in Figure 6. As shown, there was an obvious weight loss peak near 92.6 °C, attributed to the removal of adsorbed water on AC-N-SO_4_H, which was slightly lower than the 98.7 °C of AC-N but significantly higher than the 78.3 °C of AC. The results illustrated that after aqueous sulfuric acid treatment, the hydrophilicity of AC-N-SO_4_H was slightly lower than that of AC-N but still much higher than that of activated carbon. Another obvious weight loss peak in AC-N-SO_4_H appeared at 270 °C, which was mainly due to the removal of N-containing structure immobilized with sulfuric acid on the surface of carbon materials, and was slightly lower than the removal temperature of the N-containing structure on the AC-N surface at 350 °C. This was mainly caused by the introduction of the electrophilic group -SO_4_H, which reduced the stability of the N-containing structure on carbon surface. In general, after sulfuric acid immersion treatment, the surface structure stability of activated carbon was slightly worse than that of aminated activated carbon.

### 3.3. AC-N-SO_4_H Catalytic Properties in Synthesis of Acetals (Ketals)

**Figure 7:**
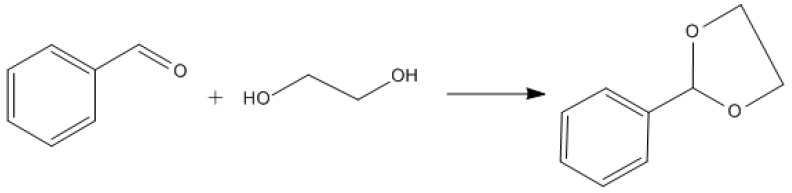
Reaction of benzaldehyde condensed with ethylene glycol.

The condensation of benzaldehyde with ethylene glycol was used as probe reaction to study the catalytic properties of AC-N-SO_4_H in the synthesis of acetals (ketals). The general procedure was as follows: 10 mL solvent cyclohexane, 25 mmol, benzaldehyde, 43.75 mmol ethylene glycol, and 0.13 g AC-N-SO_4_H were mixed in a three-necked flask equipped with a thermometer and reflux condenser and reacted 5 h at 80 °C. The effects of reaction temperature, reaction time, catalyst dosage, and molar ratio of glycol/benzaldehyde on benzaldehyde conversion were tested. At the same time, the catalytic recycling properties of AC-N-SO_4_H in reaction of benzaldehyde condensed with ethylene glycol and the applicability of different substrates were discussed. The reaction formula of benzaldehyde condensed with ethylene glycol is shown in Figure 7.

#### 3.3.1. Effects of Reaction Conditions on Benzaldehyde Conversion

**Figure 8:**
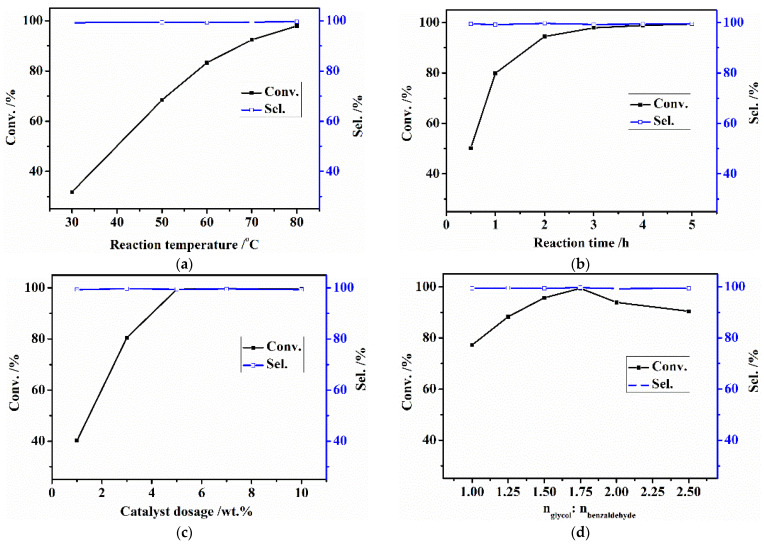
Effect of reaction conditions on benzaldehyde conversion. (**a**) Reaction temperature; (**b**) reaction time; (**c**) catalyst dosage; (**d**) molar ratio of glycol/benzaldehyde.

To determine the catalytic properties of the prepared AC-N-SO_4_H, the effects of reaction temperature, reaction time, catalyst dosage, and the molar ratio of alcohol/aldehyde on benzaldehyde conversion were discussed. The test results are shown in Figure 8a–d. According to the results, with the increased reaction temperature, reaction time, and catalyst dosage, benzaldehyde conversion increased gradually until the reaction temperature reached 80 °C, the reaction time reached 5 h, and the catalyst dosage was 5% of the benzaldehyde mass. Under the above conditions, benzaldehyde conversion was above 99%. When the molar ratio of alcohol/aldehyde was lower than 1.75, benzaldehyde conversion gradually increased with the increase of ethylene alcohol. Benzaldehyde conversion decreased to a certain extent when the amount of ethylene glycol continued to increase. The main reason for the decreasing benzaldehyde conversion was that the concentration of benzaldehyde in the reaction system was reduced with the increasing amount of ethylene glycol, which caused a decrease in collisions between molecules. The selectivity of benzaldehyde glycol acetal was above 99% under all conditions, which indicated a competitive catalysis mechanism.

#### 3.3.2. Performance of Reusability

**Figure 9:**
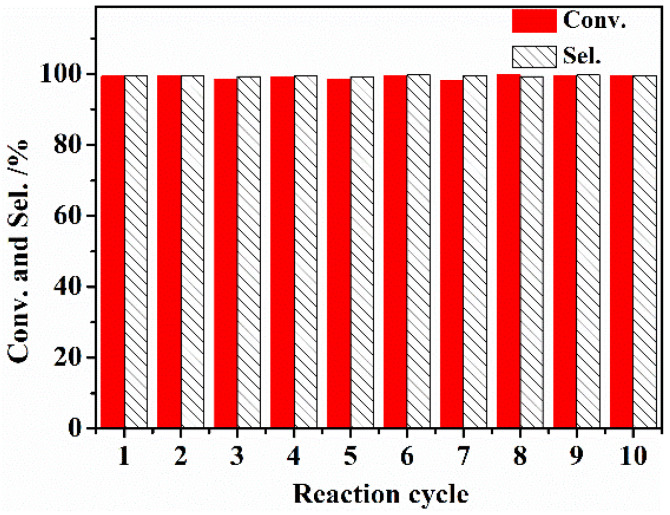
Recycling performance of AC-N-SO_4_H in benzaldehyde condensed with ethylene glycol. Note: Reaction conditions: 10 mL cyclohexane; 25 mmol benzaldehyde; molar ratio of ethylene glycol/benzaldehyde, 1.75; 0.13 g AC-N-SO_4_H; reaction temperature, 80 °C; reaction time, 5 h.

Finally, the stability of AC-N-SO_4_H was tested by performing a recycling experiment; the test results are presented in Figure 9. In the exploration of catalyst reusable performance, the catalyst AC-N-SO_4_H was washed with solvent cyclohexane and then put into the next reaction cycle. The specific process includes centrifuging out the solid after the reaction completed and washing the solid three times with cyclohexane to completely remove the small amount of residual liquid from the previous round of reaction on the surface. The performance of the catalyst showed no significant reduction even after ten successive runs, still achieving a 99% benzaldehyde conversion and 99% selectivity. Thus, AC-N-SO_4_H is an excellent and stable recyclable solid acid catalyst for the studied benzaldehyde ethylene glycol acetal reaction.

#### 3.3.3. Comparison of Catalytic Efficiency with Reported Solid Acid Catalysts

The catalytic efficiency of the prepared AC-N-SO_4_H and reported solid acid catalysts in benzaldehyde acetalization with ethylene glycol are briefly listed in Table 2. As can be seen, different kinds of solid catalysts and carbon-based solid acid catalysts modified by different methods for the reaction of benzaldehyde condensed with ethylene glycol achieved good efficiencies. Compared with the results, the AC-N-SO_4_H catalyst showed similar, sometimes even better catalytic performance under mild reaction conditions. Particularly, the prepared AC-N-SO_4_H catalyst had an advantage of good reusability, which was mainly due to the stable existence of strong acidic functional groups on activated carbon, which did not fall off in the reaction process.

#### 3.3.4. Substrate Suitability

The suitability of AC-N-SO_4_H for catalyzing synthesis of acetals (ketals) reactions with different substrates was investigated. The catalytic effects of AC-N-SO_4_H on ethylene glycol, propylene glycol, butylene glycol, chain aldehydes (ketones), cyclic aldehydes (ketones), and branched o-hydroxybenzaldehyde were investigated. The results are shown in Table 3: AC-N-SO_4_H demonstrated excellent catalytic performance on different alcohols both in chain and cyclic aldehydes (ketones). Only the conversions of salicylaldehyde with different alcohols were less than 70%, which may be due to its steric hindrance.

## 4. Conclusions

(1) With activated carbon as the raw material, a strong and stable carbon-based solid acid catalyst with hydrogen sulfate AC-N-SO_4_H with a surface acid content of 0.85 mmol/g was prepared after oxidation with HNO_3_, amination with ethylenediamine, and acidification with dilute aqueous sulfuric acid. The structural analysis showed that the specific surface area of AC-N-SO_4_H was almost the same as that of AC-N while preserving surface-active functional groups. The N-containing structure on AC-N surface was not damaged after impregnation with aqueous sulfuric acid. However, the thermal stability of the activated carbon surface structure was slightly lower than that of aminated activated carbon AC-N after sulfuric acid impregnation for introduction of the electrophilic group -SO_4_H.
(2) As a catalyst, AC-N-SO_4_H demonstrated excellent performance in synthesis of acetals (ketals) reactions. In the catalytic condensation of benzaldehyde with ethylene glycol, the conversion of benzaldehyde and the selectivity of benzaldehyde glycol acetal were both above 99%. The performance of the catalyst showed no significant reduction even after ten successive runs, still achieving a 99% benzaldehyde conversion yield and 99% benzaldehyde glycol acetal selectivity. At the same time, AC-N-SO_4_H showed excellent catalytic properties in the study of substrate applicability for the condensation reaction of ethylene glycol, propylene glycol, and butylene glycol with different chain and cyclic aldehydes (ketones), which indicated the excellent application prospects of AC-N-SO_4_H as a solid acid catalyst.
(3) The excellent catalytic properties of AC-N-SO_4_H in synthesis of acetals (ketals) can be attributed to its strong acidic functional groups and good stability. This provides a novel method for preparing carbon materials with stable strong acidic functional groups on surface. The detailed structure of the modified activated carbon surface and its catalytic mechanism still need to be further explored.

## Figures and Tables

**Table 1:** BET surface area of AC-N and AC-N-SO_4_H.

Sample	BET/m^2^·g^−1^	Pore Volume/cm^3^·g^−1^	Pore Size/nm
AC-N	418	0.26	2.5
AC-N-SO_4_H	384	0.23	2.5

**Table 2:** Comparison of the catalytic efficiency of the prepared AC-N-SO_4_H catalyst with various reported solid acid catalysts in synthesizing benzaldehyde ethylene glycol acetal.

Entry	Solid Acid Catalyst	Catalyst Amount (wt.%)	Benzaldehyde: Ethylene Glycol	Time (h)	Temp. (°C)	Conv. in the First Cycle (%)	Sel. (%)	Reaction Cycle	Conv. in the Last Cycle (%)	Ref.
1	AC-N-SO_4_H	5	1:1.75	5	80	99	100	10	99	This study
2	SulAmp-AC	3	1:3	3	90	98	-	4	92	[27]
3	GO-PrSO_3_H	3	1:3	3	90	92	-	5	80	[28]
4	Fe_3_O_4_@C-SO_3_H	1.3	1:1	2	90	69	97	9	63	[29]
5	SO_3_H/NCF-600	1.9	1:5	1	90	99	-	5	99	[30]
6	SG-[(CH_2_)_3_SO_3_H-HIM]HSO_4_	8.2	1:1.8	1.5	110	95	-	10	90	[31]
7	SulAmp-GO	3	1:3	3	90	86	-	-	-	[27]
8	CeFeTiO	6.9	1:1.6	3	110	97	-	-	-	[7]
9	[PPSH]_2_HPW_12_O_40_	5	1:1.8	3	reflux	85	-	-	-	[11]
10	HMQ-STW	7	1:3	1	105	96	100	5	90	[13]

**Table 3:** Conversion of acetals (ketals) reactions with different substrates catalyzed by AC-N-SO_4_H.

Raw Materials		Conv. (%)
Alcohol	Aldehydes (Ketones)	
Glycol	2-Pentanone	99.20
Glycol	Cyclohexanone	98.90
Glycol	Butanal	99.12
Glycol	2-Furaldehyde	96.00
Glycol	Salicylaldehyde	67.31
1,2-Propanediol	2-Pentanone	99.30
1,2-Propanediol	Cyclohexanone	99.12
1,2-Propanediol	Butanal	99.10
1,2-Propanediol	2-Furaldehyde	96.55
1,2-Propanediol	Salicylaldehyde	66.28
Butane-1,2-diol	2-Pentanone	97.26
Butane-1,2-diol	Cyclohexanone	98.09
Butane-1,2-diol	Butanal	98.73
Butane-1,2-diol	2-Furaldehyde	90.50
Butane-1,2-diol	Salicylaldehyde	63.25

Note: Reaction conditions: 10 mL cyclohexane; 25 mmol aldehyde (ketone); 43.75 mmol alcohol; AC-N-SO_4_H accounted for 5% of aldehyde (ketone) mass; reflux temperature, 5 h.

## Data Availability

The data presented in this study are available on request from the corresponding author.
